# Combustion Behavior of Sewage Sludge Hydrochar Obtained in Fast Hydrothermal Carbonization

**DOI:** 10.1021/acsomega.5c00963

**Published:** 2025-06-02

**Authors:** Guilherme Afonso de Campos Avanzi, Vinicius Sarracini Santos, Isabela Carreira Constantino, Gustavo Metzker, Mauricio Boscolo, Márcia Cristina Bisinoti, Odair Pastor Ferreira, Altair Benedito Moreira

**Affiliations:** a Institute of Biosciences, Humanities and Exact Sciences, Department of Chemistry and Environmental Sciences, 135131 São Paulo State University (UNESP), São José do Rio Preto, São Paulo 15054-000, Brazil; b Department of Chemistry, State University of Londrina, Londrina, Paraná 86055-900, Brazil

## Abstract

Sewage sludge (SS) is a complex biomass with a high content of inorganic and organic matter. The hydrothermal carbonization process (HTC) was used to produce a carbonaceous material with energy densification derived from SS. For this, temperatures of 200, 240, and 280 °C, reaction times of 30, 75, and 120 min, without or with H_2_SO_4_ acid addition (0.5 and 1% v/v) were assessed in the SS HTC process. Hydrochars (HC) were characterized by FTIR spectroscopy and CHNS elemental and thermogravimetric analyses. The energetic evaluation was performed by a higher heating value (HHV) estimation. HC presented yields ranging from 44.4 to 57.8% with a high content of organic matter and ashes due to the composition of SS. The H/C and O/C atomic ratios, ranging from 0.93 to 1.93 and 0.12 to 0.39, respectively, indicated the carbonization of SS due to dehydration and decarboxylation reactions, resulting in a carbonaceous material. FTIR analysis indicates the presence of different organic groups (*i.e*., O–H, C–H, C=C, and C=O), as well as inorganic ones (Si–O). HHV of HC ranged from 13.4 to 16.5 MJ kg^–1^ with energy densification from 0.9 to 1.1, indicating a similar energy content of HC and SS. However, energy yield efficiency ranged from 38.2 to 57.2%, being correlated with temperature and time in both acidic conditions. Combustion behavior of HC indicates that higher thermal stabilization was provided with the increase of temperature, time, and sulfuric acid.

## Graphical abstract

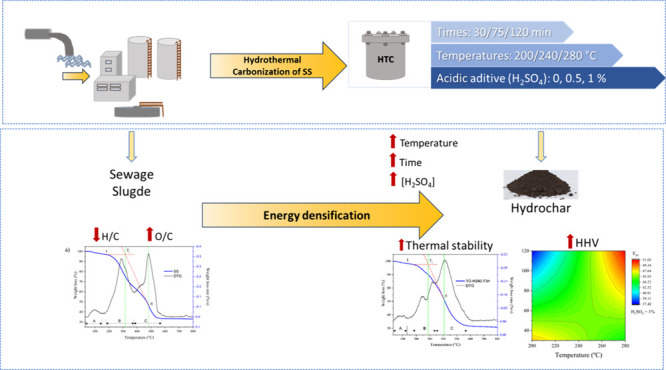

## 1 Introduction

Wastewater treatment and sludge disposal have become increasingly important topics over the past few decades. Enhancing the coverage of water treatment and promoting water reuse globally are now one of the goals within the United Nations 2030 Agenda for Sustainable Development.^1^ Currently, it is estimated that global wastewater production is approximately 359.4 × 10^9^ m^3^ yr^–1^, with only approximately52% of the wastewater being treated. This percentage varies significantly across different regions of the world.^2^ Urban wastewater consists of domestic sewage (black and gray water); water from companies and institutions (*i.e*., hospitals); industrial effluents, rainwater, and urban runoff.^3^ Therefore, with population growth coupled with urban and industrial development, effective water treatment and sustainable disposal of sewage sludge (SS) are essential.

In Brazil, the last estimated values were 12.735,92 million m^3^ yr^–1^ of household wastewater generated (sewer + septic tanks + other types of sanitation) and a production of 2.5 million tons of dry sludge per year.^4,5^ Other countries, highly populated, such as India and China, that comprise over 34% of the world population, are responsible for the generation of approximately 33 million tons of dry sludge per year.^5^ In Brazil, the most commonly used methods for wastewater treatment involve the combination of physical-chemical processes associated with Up-flow Anaerobic Sludge Blanket (UASB) reactor, which generates sewage sludge and requires treatments for disposal, such as dewatering and lime stabilization for land application, or thermal drying for landfill disposal.^6,7^ An alternative approach, which offers energy recovery, includes the UASB reactor, followed by anaerobic digestion, a cogeneration unit, dewatering, and final disposal through land application. Both methods involve high energy consumption; in this sense, new technologies that enhance the SS dewatering process are necessary.

As mentioned, millions of tons of solid residues are generated from biological treatment through the decomposition of organic matter by microorganisms. The SS consists primarily of up to 60% non-toxic organic carbon on a dry basis. Its inorganic fraction is mainly composed of silicates, aluminates, calcium, and magnesium.^8^ The inorganic composition has drawn significant interest in its application as an agricultural fertilizer, due to the agronomic properties incorporated in these substances.^9^ However, the presence in considerable concentrations of potentially toxic elements (PTE) in sewage sludge, such as toxic metals, pathogens, and organic and microbiological pollutants, has raised concerns about the potential risks associated with SS soil application.^8,10,11^

In previous studies, SS presented interesting characteristics and can be used for brick manufacture, agricultural applications,^12^ and even as adsorbents.^13^ Due to the high carbon content in SS, technologies for energy generation, or for treatment and investigation of intermediates, have been proposed, such as the generation/conversion of methane and nitrogen gases.^14–18^ Furthermore, its use for energy generation has also been evaluated in different thermochemical conversion processes of biomass, such as pyrolysis^19^ and hydrothermal carbonization (HTC), producing a solid material with high heating value, which can be applied for energetic purposes.^9,20–26^

Several studies have assessed the HTC reaction with a temperature range from 150 to 300 °C^27^ and in short reaction times, i.e., 0.25 to 24 h.^28^ Additionally, in the HTC process, the use of different substances, such as acids, bases, and salts, as additives can produce hydrochar (HC) with varying characteristics, making this process highly versatile. Acids, for instance, are particularly effective in hydrolysis of cellulose, hemicellulose, and lignin depolymerization, while also promoting other reactions, such as dehydration.^29–31^ Another advantage of HTC is that the process occurs with wet biomass, which makes the use of SS suitable and, consequently, contributes to the dewatering of this biomass and the formation of a solid phase.^32,33^ The potential of HTC to be an efficient and sustainable process for SS treatment and energy recovery has been reported. The devolatilization process results in an increase of calorific value proportional to HTC temperature, with the temperature being crucial in determining the reaction pathway and product characteristics.^34^

Therefore, this study evaluated the optimization of HTC reactions for the conversion and energy densification of SS into HC. To achieve this, short reaction times were selected to enhance the feasibility of biomass dehydration and concentration, and the effect of acid addition was also tested. Specifically, the HTC process was investigated using shorter reaction times (30, 75, and 120 min) and mild temperatures (200, 240, and 280 °C), along with the effects of H_2_SO_4_ addition (0.5 and 1.0% v/v) for the obtention of the HC. Compositional data and energy properties of the products were analyzed and correlated.

## 2 Material and Methods

### 2.1 Sewage Sludge Sampling and Hydrochar Production

The dry sewage sludge (SS) samples were collected from a sewage treatment plant located at São José do Rio Preto, São Paulo state, Brazil, and stored and frozen at −18 °C.

To produce HC, an experimental full factorial design (2^3^ + 1) was utilized, varying the HTC parameters (temperature, reaction time, and acid additive concentration) at two levels, with the addition of a central point in triplicate. All experiments were randomized to minimize systematic errors.

The HC was prepared in a stainless-steel reactor (Series 4560 mini, Parr Instrument Company, USA) using the proportion of water and SS 2:1 with temperature varying between 200, 240, and 280 °C (H200, H240, and H280, respectively) in three different times 30, 75, and 120 min (T1, T2, and T3). Sulfuric acid (98%) was used as an additive at concentrations of 0.5% and 1% v/v (named *l* and *h*, respectively), and all experiments occurred under mechanical agitation at 120 rpm.^14,15,27^ After carbonization, the solid samples were separated from the process water by filtration, and the solid was dried in an oven at 65 °C overnight. Table 1 shows the different reaction (temperature, reaction time, and acid) parameters utilized for the production of HC, as well as the respective nomenclature.

**Table 1:** Reaction Parameters Used for Each HTC

**HC sample**	**temperature (°C)**	**reaction time (min)**	**acid (% v/v)**
H200.T1	200	30	-
H200.T1*h*	200	30	1
H200.T3	200	120	-
H200.T3*h*	200	120	1
H240.T2*l*(1)	240	75	0.5
H240.T2*l*(2)	240	75	0.5
H240.T2*l*(3)	240	75	0.5
H280.T1	280	30	-
H280.T1*h*	280	30	1
H280.T3	280	120	-
H280.T3*h*	280	120	1

The HC yield (Y) was calculated from eq 1:

$$\mathrm{yield}(\%)=\frac{\mathrm{hydrochar}\;\mathrm{weight}(g)}{\mathrm{SS}\;\mathrm{weight}(g)}\times 100\%$$ 1

### 2.2 Physicochemical Characterization

Proximate analysis, thermal characteristics, and ash content of HC and SS were determined according to ASTM method D2974–14 by thermogravimetric analysis (TGA 4000, PerkinElmer, USA). The samples were heated from 30 to 800 °C at a rate of 10 °C min^–1^. For TG curves interpretation, the ignition temperature (*T_i_*) was obtained through the intersection method.^35^ Point I is the intersection of the TG curve and a vertical line passing through the first maximum peak of the DTG. At this point I, a tangent line is drawn to the TG. Point II is the beginning of devolatilization. The intersection between the straight lines passing through I and II is the ignition temperature point. The peak temperature (*T*_m_) is obtained from the identification of the maximum mass loss points on the DTG curve. The burnout temperature *T_b_* refers to the initial temperature when the mass loss rate (d*m*/d*t*) is less than 1% min^–1^.^36^ The maximum rate of loss (*R*_max_) was also determined in the DTG curve, observing the higher variation in mass loss.

The carbon, hydrogen, nitrogen, and sulfur contents were determined by elemental analysis (EA 1108 CHNS, Fisons). The percentage of oxygen was determined by eq 2:

O(%)=100%−(C+H+N+S+Ash)(%) 2

The infrared spectra (FTIR) of HC (Spectrum Two–UART Two, PerkinElmer, USA) were obtained in a spectrophotometer with an attenuated total reflectance (ATR) accessory equipped with a single reflection diamond crystal (ATR-FTIR). The solid material was placed directly on the ATR crystal and analyzed in a range of 4000–400 cm^–1^ and 30 scans.

### 2.3 Higher Heating Value (HHV) Determination

The higher heating values (HHV) of HC and SS were estimated by the Dulong equation (eq 3) which correlates the HHV of solid fuel with the material’s elemental composition.^37,38^

$$\mathrm{HHV}=(0,338\times C)+[1,428\times (H-\frac{O}{8})]$$ 3

where C, H, O, and S are the dry basis mass percentages of the carbon, hydrogen, oxygen, and sulfur contents, respectively.

The energy densification (*E*_*D*_) and energy yield efficiency (*E*_*ye*_) were calculated using eqs 4 and 5:

$$E_{D}=\frac{\mathrm{HHV}\;\mathrm{of}\;\mathrm{hydrochar}}{\mathrm{HHV}\;\mathrm{of}\;\mathrm{dry}\;\mathrm{SS}}$$ 4

$$E_{ye}(\%)=E_{D}\times Y\times 100\%$$ 5

## 3 Results and Discussion

### 3.1 Dry Sewage Sludge and Hydrochars Characterization

The yield, ash content, organic matter, and CHNS elemental analysis of SS and HC are listed in Table 2. The HC yield varied from 44.4 to 57.8%, organic matter (OM) from 42.2 to 54.6%, and ash content ranged from 34.2 to 53.7%. The HC yield did not show a clear trend with increasing temperature and time, possibly due to the high content of ash in the material. Ash content exhibited an increasing trend with higher temperature and longer reaction times, conversely, OM presented a slightly decreasing trend.

**Table 2:** Comparison of Yield, Ash, Organic Matter, and Elemental Composition of Sewage Sludge (SS) and Hydrochars (HC) of This Study with Data from Other Sewage Sludge Hydrochar Studies in the Literature^d^

		**(%)**								**atomic ratio**	
**samples**		**yield**	**ash**	**OM**	**C**	**H**	**N**	**S**	O*	**H/C**	**O/C**
present study	SS	-	34.2	65.8	39.5	4.1	nd	2.0	20.6	1.24	0.39
	H200.T1	57.4	48.8	51.2	32.2	4.5	2.4	1.6	10.4	1.67	0.24
	H200.T1*h*	48.8	45.4	54.6	32.6	4.4	3.9	0	13.7	1.61	0.32
	H200.T3	46.1	48.9	51.1	30.9	4.6	2.0	1.4	12.2	1.77	0.30
	H200.T3*h*	44.4	50.2	49.8	33.2	2.6	2.4	2.8	8.8	0.93	0.20
	H240.T2l**	50.3 ± 6.2	50.8 ± 3.2	49.2 ± 3.2	34.0 ± 2.5	3.6 ± 0.7	1.4 ± 1.2	3.7 ± 0.3	6.6 ± 0.8	1.26	0.15
	H280.T1	51.0	57.8	42.2	31.6	3.4	0	2.1	5.1	1.28	0.12
	H280.T1*h*	50.4	49.7	50.3	34.2	2.8	0	3.9	9.3	0.98	0.20
	H280.T3	57.8	53.7	46.3	30.2	4.7	2.0	1.1	8.3	1.85	0.21
	H280.T3*h*	46.4	51.5	48.5	31.1	5.0	1.7	4.4	6.3	1.92	0.15
previous studies	HC^a^	-	5.7	-	67.2	4.3	0.04	0.0	22.8	0.76	0.25
	HC^b^	72.9	-	58.2	38.4	5.2	2.7	-	11.9	1.61	0.23
	HC^b^	56.8	-	58.4	40.1	5.2	2.4	-	10.7	1.54	0.20
	HC^c^	-	22.8	-	41.3	4.9	4.5	2.9	12.8	1.41	0.23

a Costa et al. 2019 (HC from babassu mesocarp – 180 °C/48h/pH 3);^41^

b Gaur et al. 2020 (HC from SS - 200 °C/30 min);^38^ ^b^Gaur et al. 2020 (HC from SS - 200 °C/120 min);^38^

c Malgorzata et sl. 2023 (HC from SS – 200 °C/120 min/pH 2).^29^

d (−) Not applicable; abbreviations: OM, organic matter; TOC, total organic carbon, nd: not detected; (*)­O(%) = 100% – (C + H + N + S + Ash); (**) mean ± deviation of triplicate (%).

These results can indicate a gasification of volatile matter and conversion of inorganic material by retention of minerals^34,39^ corroborating that higher temperatures and long reaction times promote greater carbon fixation and the elimination of volatile matter, resulting in decreasing OM.^20,24,27,40^ The relatively high ash content of HC is a result of the loss of volatile matter, dissolution of organic compounds in the liquid phase, and precipitation of inorganic compounds on the surface of the HC.^39^

HC obtained under acidic conditions also showed an increase in carbon content compared to conditions without sulfuric acid. The HTC reactions at acidic conditions lead to an increase in particle plasticity, which consists of lower energy for compression and larger particle size.^42^ Previous studies indicated that HC synthesized from Babassu mesocarp in acidic conditions promotes a more effective carbonization than in basic conditions, indicating higher carbon fixation in the solid structure at a similar temperature.^41^

The oxygen content in HC decreased from 20.61% in SS to values close to 5.08% at 280 °C. As expected, in HTC, this reduction corroborates the formation of solid carbonaceous products with lower oxygen content through decarboxylation and dehydration, resulting in a more hydrophobic structure.^22,23,36,41^

A reduction in nitrogen content is expected during HTC, which is attributed to gasification reactions or transfer to the liquid phase due to the hydrolysis of structural amino acids from proteins present in SS. However, HC exhibited higher nitrogen content than SS, resulting from the concentration of nitrogen compounds during HTC, leading to an HC with a proportionally higher nitrogen content.^40^

The degree of carbonization can be indicated through H/C and O/C atomic ratios. The H/C atomic ratios ranged from 0.93 for H200.T3*h* to 1.92 for H280.T3*h*, while SS had a ratio of 1.24. Regarding the O/C atomic ratio, a decrease was observed for all HC samples compared to SS (0.39), whose values ranged from 0.12 for H280.T1 to 0.31 for H200.T1*h*. Studies have shown that a high O/C atomic ratio indicates materials with a low degree of carbonization and oxygen group content. Furthermore, these authors concluded that H/C > 1 suggested that the HTC does not promote the formation of highly condensed aromatic structures, indicating greater evidence of aliphatic groups.^39^

Previous studies on hydrochar from sewage sludge at 200 °C for 30 and 120 min showed a decrease in yield with increasing reaction time; nevertheless, the H/C and O/C atomic ratios remained similar.^38^ This behavior was also observed in our study under conditions without sulfuric acid at 200 °C (H200.T1 and H200.T3), where these ratios did not show considerable changes. However, upon comparison of the conditions, the presence of sulfuric acid at 200 °C and 120 min resulted in a decrease in the H/C ratio from 1.77 to 0.98, indicating an increase in the aromaticity of hydrochar.

The FTIR spectra of the produced HC and SS are presented in Figure 1. The intense band at 3400 cm^–1^ is particularly attributed to the O—H stretching vibration of hydroxyl groups present mainly in SS and disappearing in all HTC conditions performed, indicating dehydration reactions and corroborating with elemental analysis. The band between 3000 and 2800 cm^–1^ is a region that corresponds to the C—H bonds of methyl and methylene groups, attributed to aliphatic CH_n_ groups,^36^ can be seen in all conditions, except for the temperature of 280 °C in 30 min, and may indicate the formation of aromatic groups in this condition. The lower intensity of this band in the mentioned condition can also be explained due to the higher ash contents in the HC, around 57.8%, the highest found in our study.^20^

**Figure 1:**
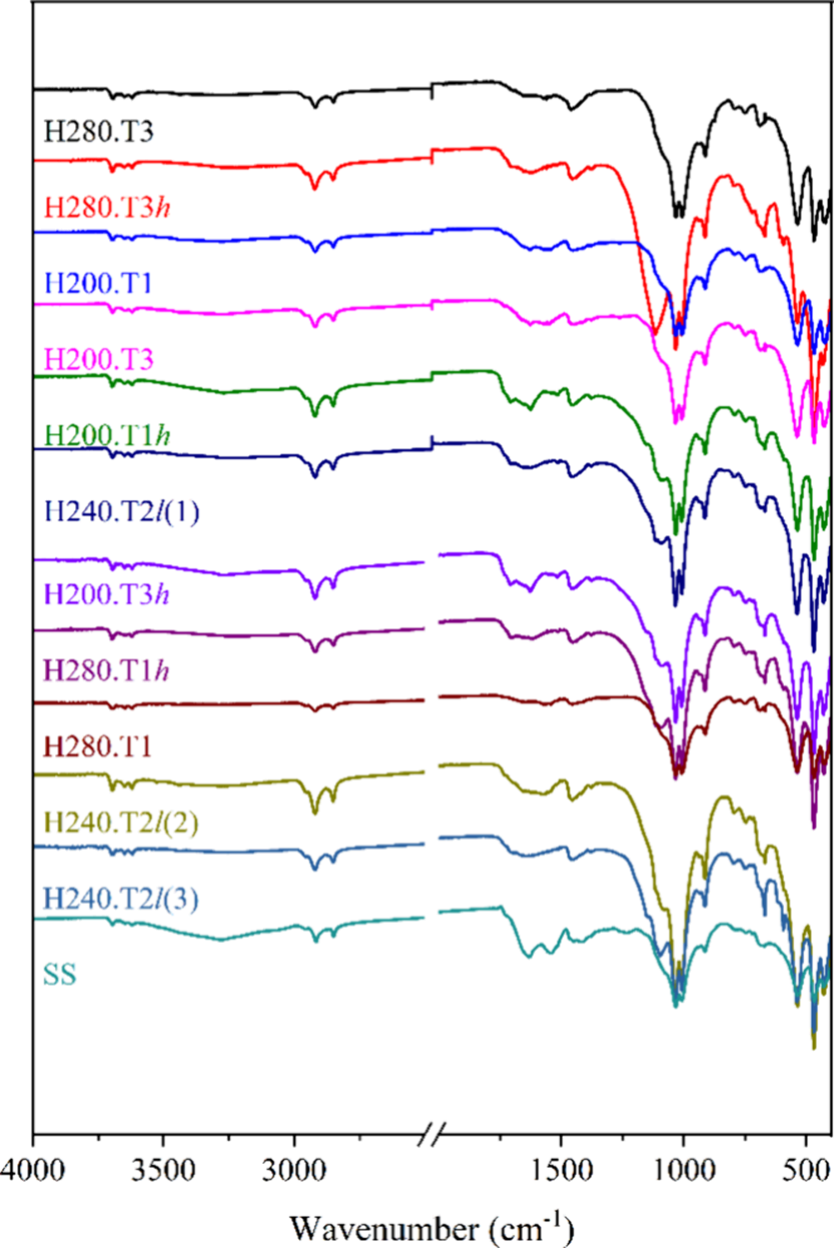
FTIR spectra of SS and hydrochars.

A band attributable to hydrophobic C=O groups can be visible around 1620 cm^–1^ for all samples except for H280.T1, suggesting that there were no condensation and aromatization reactions in this condition, in addition to high ash content.^20^ A band near 1540 cm^–1^ was identified in SS, attributed to asymmetric stretching of —C=O bonds in carboxylic groups, and also the band at 1455 cm^–1^, attributed to C—C=C stretching in aromatic ring carbons, which tend to increase with time and temperature in HTC.^22^ Overall, all spectra also show bands around 1700 cm^–1^, associated with the —C=O group in saturated carboxylic acids, which may indicate splitting of glycoside rings in the chains and generating aliphatic chain structures.^41,43^ The presence of this range of oxygenated groups suggests the decarboxylation reactions that alter these groups as reaction time increases.^23^

The bands with between 800 and 1200 cm^–1^ highlight inorganic content in the material,^20^ especially the bands around 1000 cm^–1^, which can be attributed to the Si—O stretching vibration that suggests the presence of silicates in SS and its HC.^36^

### 3.2 Combustion Behavior and Thermal Characteristics of Hydrochars

The higher heating values, energy densification, and energy yield efficiency of HC are presented in Table 3.

**Table 3:** Higher Heating Values (HHV), Energy Densification (*E_D_*), and Energy Yield Efficiency (*E_ye_*) of SS and Hydrochars

	**HHV (MJ kg** ^**–1**^ **)**	**HHV/OM (MJ kg** ^**–1**^ **)**	***E**_**D**_*	***E**_**ye**_* **(%)**
SS	15.5	23.6	1.0	
H200.T1	15.4	30.9	1.0	57.1
H200.T1*h*	14.9	27.2	1.0	46.1
H200.T3	14.8	29.0	1.0	44.0
H200.T3*h*	13.4	26.6	0.9	38.2
H240.T2*l* ^a^	15.4 ± 1.9	31.2 ± 1.9	1.0 ± 0.1	49.9 ± 9.2
H280.T1	14.6	34.7	0.9	47.8
H280.T1*h*	13.9	27.6	0.9	45.9
H280.T3	15.4	33.3	1.0	57.2
H280.T3*h*	16.5	34.1	1.1	49.7

a Mean ± deviation of triplicate.

For HC, conditions with higher temperatures presented higher HHV (280 °C); however, it is necessary to consider the ash contents, the HHV/OM index being adequate to indicate the energetic content resulting from the organic fraction of HC. This index also corroborates with HHV, indicating that higher temperatures (280 °C) promote the production of HC with higher energetic content. Studies conducted on the effect of HTC parameters on fuel properties of SS-derived HC concluded that an increase in temperature improves the fuel properties of carbonized sewage sludge.^44^

To correlate the HHV of HC with reaction yields, *E_ye_* is an important parameter to consider. Figure 2 shows the contour plot for energy yield efficiency (*E_ye_*) for the HC under conditions without sulfuric acid and with the addition of 1% sulfuric acid.

**Figure 2:**
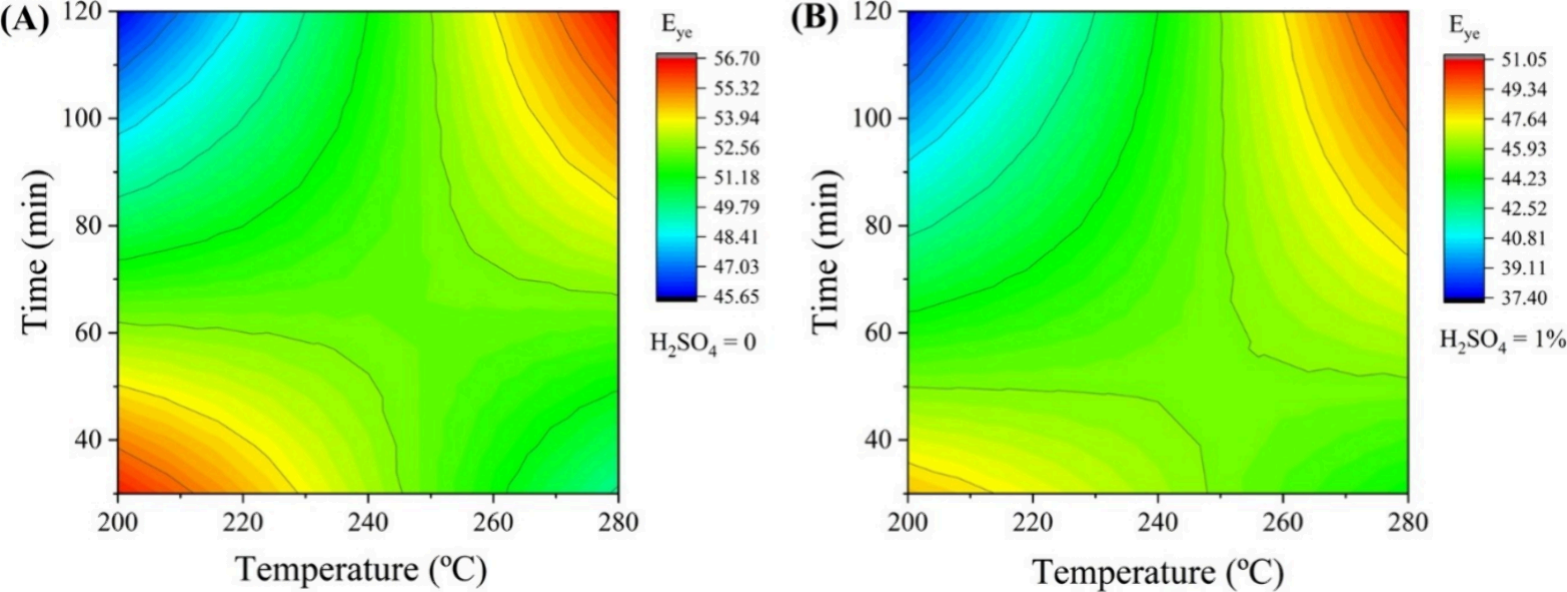
Energy yield efficiency of hydrochars in different times and temperatures: (A) without an acidic additive and (B) with 1% (v/v) H_2_SO_4_.

Considering the *E_ye_*, which includes the HHV and reaction yield, conditions without H_2_SO_4_ (Figure 2A) at lower temperatures and reaction times (200 °C, 30 min) showed higher *E_ye_* due to the lower degradation of SS, resulting in a higher yield (%), as observed in Table 2. On the other hand, lower temperatures and reaction times (200 °C, 30 min) with 1% H_2_SO_4_ presented low *E_ye_*, possibly due to the dehydration characteristics of sulfuric acid, which reduced the yield of reaction (Figure 2B).

At higher temperatures and longer reaction times (280 °C, 120 min), in both acidic conditions, a higher *E_ye_* can be observed with the increasing the HHV of HC, as discussed in the context of densification of energy.^45^ Additionally, low temperatures with high reaction times (200 °C, 120 min) resulted in lower *E_ye_* under both conditions due to reduced yield and lower HHV.

Previous studies of HTC of SS at different pH values using sulfuric acid at 200 °C for 120 min observed that, at low temperatures, the acidic additive increases the HHV compared to SS. However, as the acidity increases, the HHV decreases from 19.04 to 18.72 MJ kg^–1^, under conditions without sulfuric acid and at pH 2, respectively.^29^ This behavior corroborates the idea that temperature is an important parameter for increasing the HHV of HC, as described in the literature.^23^

The HHV obtained for the HC was approximately 15 MJ kg^–1^, similar to that observed for lignite.^22^ Studies utilizing SS with different waste biomass (sawdust, corncob, cornstalk, and rape straw) for char production obtained HHV values of 9.64 MJ kg^–1^ at 220 °C 10.11 MJ kg^–1^ at 260 °C, and 10.32 MJ kg^–1^ at 300 °C. These results are lower than those observed in this study, even at higher temperatures.^46^

For HC production of SS with sawdust and cornstalk, the HHV values reported g ranged from 13.61 to 16.79 MJ kg^–1^, being similar to those found in this study.^47^ Other studies have also produced HC derived from SS, obtaining *E_D_* values between 1.01 and 1.12 under an oxygen-free atmosphere inside the reactor.^33^ In our study, HTC reactions conducted under ambient atmosphere presented a similar E_D_ for H280.T3h (*E_D_* = 1.1), providing an advantage over the mentioned study, due to the lack of need for pressure generated by gases, such as N_2_.

The TG and derived thermogravimetry (DTG) curves (Figure S1), illustrate the combustion behavior of HC and SS. The processes that occurred in the material were divided into three stages, A, B, and C, corresponding to the dehydration phase, oxidative pyrolysis/devolatilization phase, and combustion/oxidation phase, respectively.^35,48^

In the first stage A, moisture release and a small mass loss occurred due to the evaporation of water and the release of some low molecular mass volatiles at temperatures ranging from 30 to 200 °C.^48^ In stage B, hemicellulose and cellulose decompose, with the beginning of lignin decomposition, resulting in significant mass loss.^49^ Stage C (350–600 °C) consists of oxidation reactions of materials formed in the second stage, along with the decomposition of complex structures, aliphatic or aromatic, such as the residual lignin and sugars.^11,49^

The SS showed intense DTG peaks in B and C, indicating decomposition reactions, depolymerization, and oxidation of organic matter, with their thermal events ending near 600 °C. The SS underwent devolatilization with a maximum DTG peak between 179 and 382 °C, differing from HC at temperatures ranging from 187 to 349 °C. The end of this thermal event at a lower temperature suggests a lower volatile matter content in the carbonized products, indicating greater carbon fixation in the structure, similar to results in the literature.^36^ The H280.T1*h* and H280.T3*h* samples exhibited their most intense DTG peaks in the C phase of combustion, between 348 and 545 °C and 345 and 576 °C, respectively, indicating higher oxidative degradation. This behavior contrasts with the HC processed at 200 °C, which showed higher mass losses in phase B or similar losses in both phases B and C. The *T*_i_, *T*_m_, *T*_b_, and the maximum mass loss rate *R*_max_ for all hydrochars are presented in Table 4.

**Table 4:** Temperature Characteristics of the Pyrolysis and Combustion Behavior of Hydrochars and SS

sample	*T*_i_ (°C)	*T*_m_ (°C)	*T*_b_ (°C)	*R*_max_ (%/min)
SS	236	288	574	3.0
H200.T1	263	296	561	3.4
H200.T1*h*	275	461	550	2.3
H200.T3	261	299	543	3.4
H200.T3*h*	283	463	538	2.2
H240.T2*l* ^a^	271 ± 12.75	367 ± 55.84	548 ± 10.30	2.6 ± 0.2
H280.T1	255	303	547	2.0
H280.T1*h*	300	415	545	2.6
H280.T3	246	310	555	2.1
H280.T3*h*	288	407	576	2.3

a Mean ± deviation of triplicate.

Among the HC, the lowest T_i_ were observed for H280.T3 and H280.T1, which were 246 and 255 °C, respectively, indicating a less stable combustion process in these conditions.^50^ The highest *T*_m_ was observed in condition H280.T1*h*, indicating that sulfuric acid in the HTC process led to the production of HC with greater combustion stability.

The *T*_m_ results highlight the effects of the increased severity of HTC reactions on the HC that is produced. With the same temperature range, the addition of acid promoted an approximately 55% increase in *T*_m_, in HTC at 200 °C. Smaller variations were observed at the highest HTC temperature (280 °C), 37% for 30 min reactions and 31% for 120 min reactions. As observed in T_i_, which suggests combustion stabilization, sulfuric acid influenced the *T*_m_ by increasing the temperature of reactivity (or reactivity point) of HC.^51^ This increase in *T*_m_ has also been observed in previous studies utilizing SS and Camellia oleifera shells.^50,51^

The *T*_b_ under all HTC conditions was obtained near 550 °C, indicating a lower relative mass loss during the process. It is possible to observe the constant mass after this temperature. This behavior differs from that of SS, in which the temperature of the TG curve stabilizes around 600 °C, associated with a high volatile matter content and low fixed carbon content. Previous studies associate the loss of organic matter up to 600 °C, while a slight relative mass loss between 600 and 700 °C has been associated with the inorganic material present in the HC and biomass.^11^

With the increase of temperature, time, and sulfuric acid, the maximum mass loss rate (*R*_max_) presented lower values, corroborating with *T*_i_ and *T*_m_ values, which indicates the higher stabilization of organic matter, increasing the temperature of reactivity (or reactivity point) of HC. Previous studies of HTC of SS found an *R*_max_ of 3.84 in SS and 1.74 for HC obtained from this biomass, indicating a trend similar to that observed in this study. This rate is lower than that observed for lignocellulosic materials, such as bagasse.^52^

## 4 Conclusions

Hydrothermal carbonization was effective in transforming sewage sludge into HC, presenting a high content of organic matter and ashes, as evidenced in elemental analysis and FTIR results. The energetic densification was observed for all hydrochars, which showed a higher HHV/OM index compared to the precursor biomass (SS), especially the hydrochars produced under conditions with higher temperatures, longer times, and higher sulfuric acid concentration (H280.T1, H280.T3, and H280.T3*h*). These HTC conditions also improved the higher energy yield (*E_ye_*). Combustion behavior also corroborated these findings, which indicated that acidic sulfuric conditions, as well as higher temperature and time, produced a more stable HC for energy purposes. Therefore, this study broadens the perspective for HC production from SS, showing that the conversion of SS to HC can be improved by additives during the hydrothermal process.

## Supplementary Material



## Data Availability

The data is available at the following address: https://hdl.handle.net/11449/310301.

## Notes

TG and DTG curves of all hydrochars (PDF)
