# Supplementary material for: Combustion Behavior of Sewage Sludge Hydrochar Obtained in Fast Hydrothermal Carbonization
Source: ACS Omega. 2025 Jun 2;10(23):24502–9. doi: 10.1021/acsomega.5c00963 (PMC12177602; doi:10.1021/acsomega.5c00963)
Supplement: Supplementary file 1 [file ao5c00963_si_001.pdf]

COMBUSTION BEHAVIOR OF SEWAGE SLUDGE HYDROCHAR OBTAINED IN FAST HYDROTHERMAL CARBONIZATION

Guilherme Afonso de Campos Avanzi[a], Vinicius Sarracini Santos[a], Isabela Carreira Constantino[a], Gustavo Metzker[a], Mauricio Boscolo[a], Márcia Cristina Bisinoti[a], Odair Pastor Ferreira[b], Altair Benedito Moreira[a*]

[a] São Paulo State University (UNESP), Institute of Biosciences, Humanities and Exact Sciences, Department of Chemistry and Environmental Sciences, Zip code 15054-000, São José do Rio Preto, São Paulo, Brazil

[b] State University of Londrina, Department of Chemistry, Zip code 86055-900, Londrina, Paraná, Brazil.

**Corresponding author*
e-mail: altair.moreira@unesp.br
Phone: +55 17 3221-2509

Supplementary material

Figure S1 – TG–DTG curves of SS and hydrochars derived from sewage sludge: (**a**) SS, (**b**) H200.T1, (**c**) H200.T1*h*, (**d**) H200.T3, (**e**) H200.T3*h*, (**f**) H240.T2*l* (1), (**g**) H240.T2*l* (2), (**h**) H240.T2*l* (3), (**i**) H280.T1, (**j**) H280.T1*h*, (**k**) H280.T3 and (**l**) H280.T3*h*.

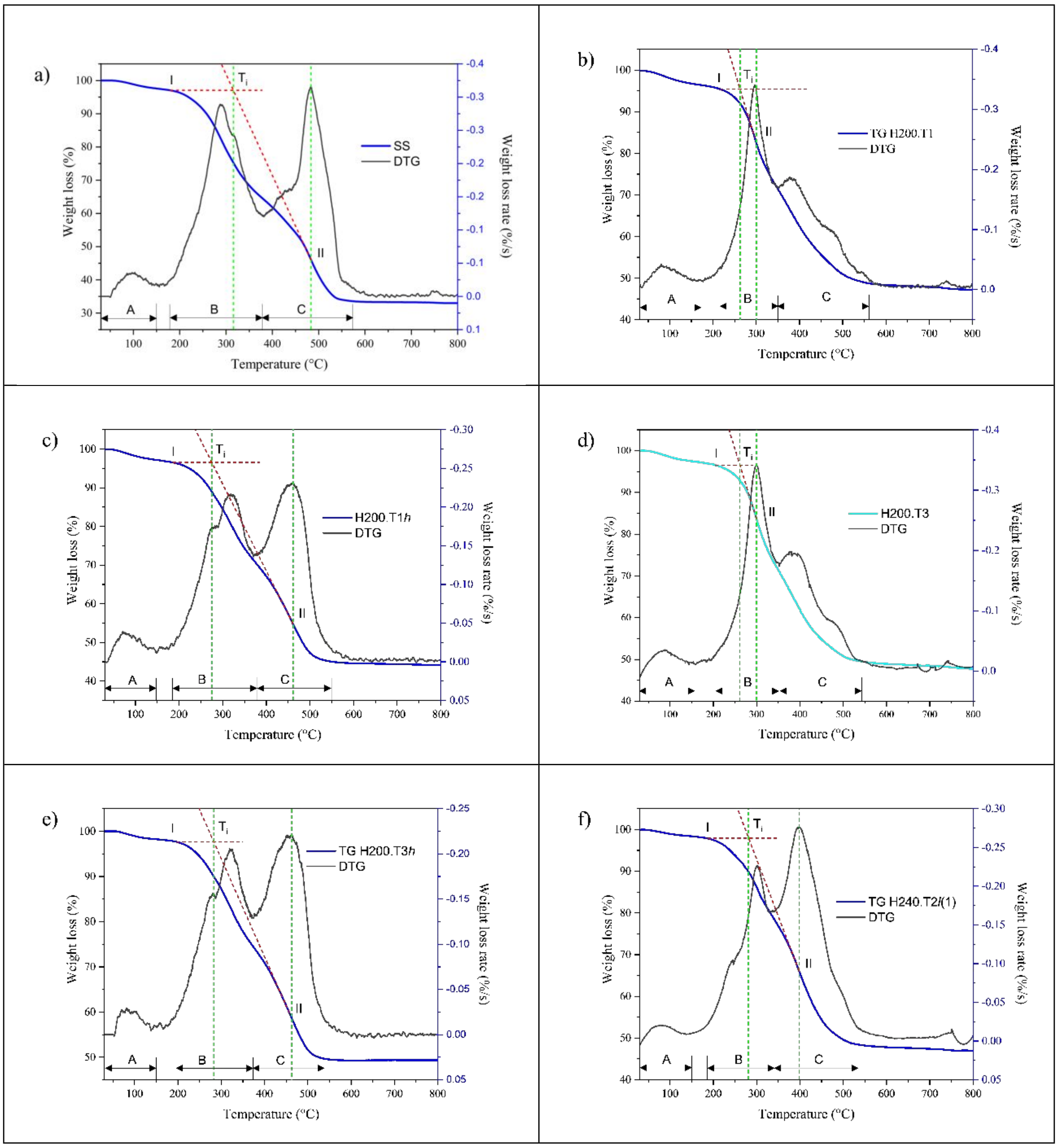

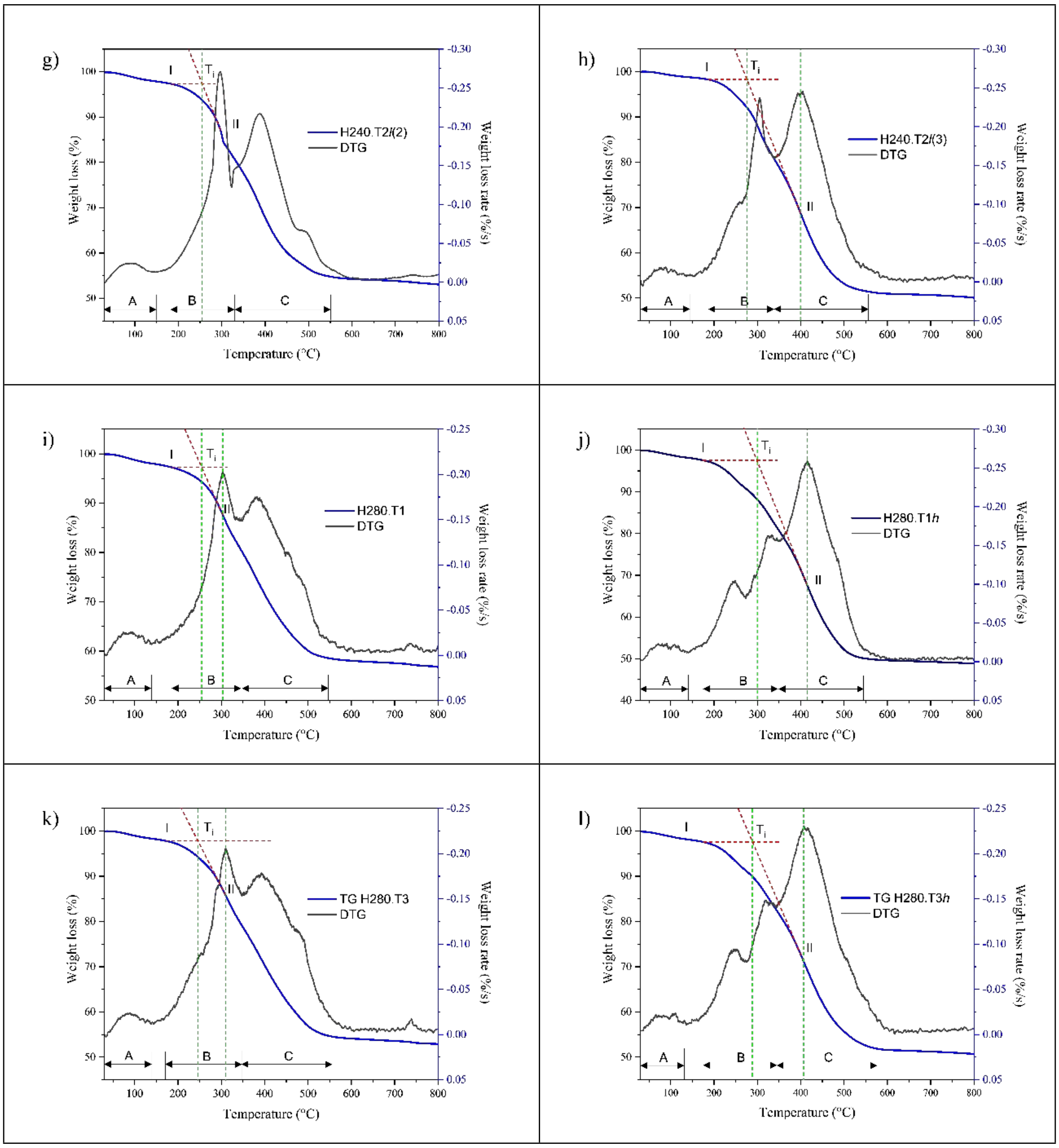

g)
H240.T2l(2)
DTG
h)
H240.T2l(3)
DTG
i)
H280.T1
DTG
j)
H280.T1h
DTG
k)
TG H280.T3
DTG
l)
TG H280.T3h
DTG
I
II
T_i
A
B
C
Weight loss (%)
Weight loss rate (%/s)
Temperature (°C)
